# Effects of Post-Curing Time on the Mechanical and Color Properties of Three-Dimensional Printed Crown and Bridge Materials

**DOI:** 10.3390/polym12112762

**Published:** 2020-11-23

**Authors:** Dohyun Kim, Ji-Suk Shim, Dasun Lee, Seung-Ho Shin, Na-Eun Nam, Kyu-Hyung Park, June-Sung Shim, Jong-Eun Kim

**Affiliations:** 1 Department of Conservative Dentistry, Yonsei University College of Dentistry, Yonsei-ro 50-1, Seodaemun-gu, Seoul 03722, Korea; dohyun0kim@yuhs.ac (D.K.); leedasun@yuhs.ac (D.L.); 2 Department of Dentistry, Korea University Guro Hospital, Guro-gu, Seoul 08308, Korea; shoss@daum.net; 3 Department of Prosthodontics, Yonsei University College of Dentistry, Yonsei-ro 50-1, Seodaemun-gu, Seoul 03722, Korea; shin506@prostholabs.com (S.-H.S.); jennynam90@yuhs.ac (N.-E.N.); khyungpark@yuhs.ac (K.-H.P.); jfshim@yuhs.ac (J.-S.S.)

**Keywords:** additive manufacturing, three-dimensional printing, rapid prototyping, post-curing, CAD/CAM, flexural strength, Weibull modulus, degree of conversion, Vickers hardness

## Abstract

Three-dimensional (3D) printing is increasingly being utilized in the dental field. After fabricating a prosthesis using a 3D printed resin, a post-curing process is required to improve its mechanical properties, but there has been insufficient research on the optimal post-curing conditions. We used various 3D printed crown and bridge materials in this study, and evaluated the changes in their properties according to post-curing time by evaluating the flexural strength, Weibull modulus, Vickers hardness, color change, degree of conversion, and biocompatibility. The obtained results confirmed that the strength of the 3D printed resin increased when it was post-cured for 60–90 min. The Vickers hardness, the degree of conversion, and biocompatibility of the 3D printed resins increased significantly around the beginning of the post-curing time, and then increased more gradually as the post-curing time increased further. It was observed that the color tone also changed as the post-curing time increased, with some groups showing a ΔE_00_ value of ≥ 2.25, which can be recognized clinically. This study has confirmed that, after the printing process of a 3D printed resin was completed, a sufficient post-curing time of at least 60 min is required to improve the overall clinical performance of the produced material.

## 1. Introduction

Recent developments in digital technologies applied in the dental field are increasing the proportion of prostheses produced using computer-aided design/computer-aided manufacture (CAD/CAM) systems, thereby gradually replacing traditional methods [1]. In the scanning stage, after a tooth abutment or implant placement has been prepared, the definitive cast produced through impression-taking is scanned using a model scanner and then a virtual model is created, or else the oral cavity is directly digitalized using an intraoral scanner [2,3]. Dentists and dental technicians use the obtained information to design the prosthesis using dental CAD software, and manufacture the prosthesis by milling or three-dimensional (3D) printing [4,5]. This approach minimizes various kinds of errors that can occur in the traditional method used to manufacture a prosthesis, and also makes the overall manufacturing process more efficient [6].

The methods used to produce a prosthesis designed with CAD software are divided into subtractive and additive methods. Subtractive processing uses a milling machine to cut a prefabricated disk or block-shaped materials. In contrast, the additive method, which is also known as 3D printing or rapid prototyping, involves creating a desired shape by printing materials, such as photocurable resins or thermoplastic filaments, in a layer-by-layer manner. The milling accuracy in the subtractive method is affected by both the characteristics of the material and the milling tool used during manufacturing, with some materials being difficult to mill due to characteristics such as their hardness or elasticity [7]. The subtractive method has the additional disadvantage of it being difficult to manufacture a complex shape, and the drill and bur used for this process wear out over time, which may lead to the fabrication of inaccurate prostheses [8,9,10]. In contrast, the additive method does not require the use of a drill or bur, which avoids any problems associated with abrasion, and it is possible to produce a more-complex shape or design such as hollowing out the internal structure. This potentially makes the additive method more suitable for the dental field [8]. The additive method also avoids unnecessary waste due to only the required part needing to be manufactured, which provides an economic advantage over the milling method [7]. The most popular additive prosthesis manufacturing methods are stereolithography (SLA), digital light processing (DLP), and fused-deposition modeling. SLA and DLP 3D printers have the advantages of high accuracy and rapid processing, and are often used to manufacture prostheses [11,12]. The types of materials and ingredients of 3D printed resins used in dentistry are very diverse, and the shade of 3D printed resins for crowns and bridges can also cover a very diverse range to suit the variety of shades of natural teeth [5,13].

Various parameters need to be controlled in the 3D printing process. The mechanical and physical properties of the printing material are affected by the thickness of each printed layer, the depth and degree of polymerization, shrinkage between the layers, and the intensity and angle of the irradiation light source [14,15,16,17]. The direction of 3D printing also affects the mechanical strength [15,18,19]. Even when printing the same shape, the number of layers may vary depending on the printing direction, and shrinkage between layers may occur [17]. It is very important to fully understand the various parameters in 3D printing that affect the mechanical strength of a printing material, and can therefore also affect the quality or strength of the produced device or prosthesis [14,20].

After printing, the photopolymerization resin is additionally subjected to a post-curing process in an ultraviolet (UV) oven to improve its mechanical strength by polymerizing unreacted monomers while ensuring that the polymerization is even and complete in all regions [8,21,22]. The duration of this post-curing process differs for each company that produces photopolymerized resins and for each type of material, and it can even differ markedly with the 3D printing equipment and post-curing equipment used [23,24]. Previous studies have found that the mechanical strength as well as the accuracy can be affected by the post-curing process [20,25], and the use of different post-curing equipment results in different post-curing outcomes [23,24].

Previous studies of the differences in polymerization rates or color changes according to the post-curing time have been insufficient, which makes it necessary to further investigate how the type of post-curing equipment and post-curing time influence the final product. In particular, 3D printed crown and bridge materials are mainly used for temporary restorations. Therefore, it is very important to produce a prosthesis with optimal mechanical properties and an appropriate color tone when a temporary restoration is used for a long time in orthodontic treatment or to produce alterations in vertical dimensions, or if the patient has a nonfunctional habit [26]. It is extremely important to evaluate the biocompatibility of the 3D-printed prostheses. This is because the 3D printed prostheses may have different results depending on the parameters of the printing, washing, and post-curing processes, unlike subtractive prostheses made by processing blocks of prefabricated materials [27]. Knowledge of how the characteristics of 3D printed objects vary with the post-curing time could provide very meaningful guidelines for clinicians and dental technicians who manufacture prostheses.

This study evaluated how the mechanical and color characteristics of various crown and bridge materials that are available commercially vary with the post-curing time. The null hypothesis of this study was that the flexural strength, hardness, color tone, degree of conversion and biocompatibility of 3D printed crown and bridge photopolymerized resins do not differ with the post-curing time.

## 2. Materials and Methods

### 2.1. Specimen Preparation

**Figure 1:**
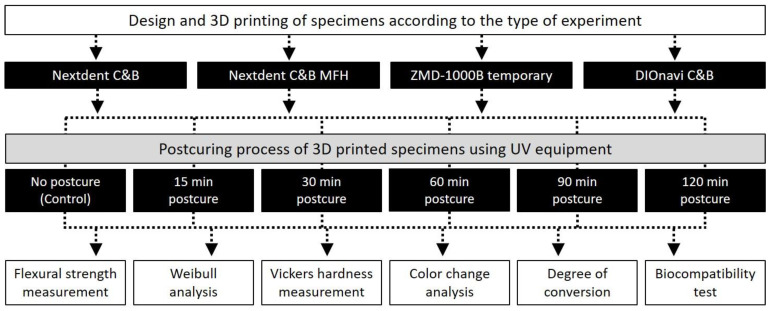
Flowchart of the overall experimental process of this study, showing the materials used, post-curing time, and experimental types.

The overall experimental process of this study is depicted in detail in Figure 1.

Four types of 3D printed crown and bridge materials were used in this study: Nextdent C&B (Nextdent, Soesterburg, The Netherlands), Nextdent C&B MFH (Nextdent, Soesterburg, The Netherlands), ZMD-1000B temporary (Dentis, Daegu, Korea), and DIOnavi C&B (DIO Incorporated, Busan, Korea). The properties of these materials used are reported in Table 1.

Each specimen was designed using Rhino 6 software (Robert McNeel & Associates, Seattle, WA, USA). The specimen used for evaluating the flexural strength was constructed as a bar-like shape with a width and thickness of 2 mm and a length of 25 mm, referring to DIN EN ISO 4049 (dental polymer-based materials) [28]. The design file of the specimen was exported to a file in the STL (standard tessellation language) format, and then the process of arranging the support configuration on the building platform and 3D printing proceeded using 3D printing slicer software (VeltzBP, Hebsiba, Seoul, Korea). The printing orientation was determined to be 0 degrees for the build platform, based on the results of accuracy evaluations of the thickness, width, and length according to the printing orientation obtained by Tahayeri et al. [15]. The thickness of each printed layer was set to 100 μm, and 20 specimens were produced per group.

3D printing of the specimens for the Nextdent C&B, Nextdent C&B MFH, and DIOnavi C&B resins was performed using a Veltz D2 3D printer (Hepsiba, Seoul, Korea), with the parameters for each 3D printed resin adjusted in a pilot study. For the ZMD-1000B temporary resin, specimens were prepared using a Zenith D 3D printer (Dentis). In all 3D printing processes, lamination was performed with a thickness of 100 µm and photocuring was performed for 20 s per layer. A 405-nm UV LED light with an intensity of 1.4 mW/cm^3^ was used by the 3D printer. When producing the specimens, a single bottle of resin was used for each material in order to avoid variations between specimens, and the resin tank that was used for each material had never been used before. The monomer remaining on the surface of the specimen was washed off using 90% isopropanol.

The post-curing process was carried out using a post-curing equipment (CureM D102H, SONA Global, Seoul, Korea), with a UV intensity of 220 µW·cm^−2^ and an internal temperature of 60 °C. The green-state group (without post-curing) was used as a control, and post-curing times of 15, 30, 60, 90, and 120 min were applied in the other groups. After post-curing, the support structure used for printing was removed. The specimens in the green-state group were dried after washing with alcohol. After the post-curing process was completed, the length, width, and thickness of the specimen were confirmed to be 25 mm, 2 mm, and 2 mm, respectively. The dimensions of the specimens were measured using a high-precision digital caliper (Mitutoyo Manufacturing Co. Ltd., Tokyo, Japan) with an accuracy of 0.01 mm.

### 2.2. Flexural Strength Test and Weibull Analysis

The flexural strength of the specimens was determined using a three-point bending test, which was implemented using a universal testing machine (Model 3366, Instron Corporation, Norwood, MA, USA) equipped with a 10-kN load cell and at a crosshead speed of 0.75 mm·min^–1^, in accordance with ISO 4049. Before each measurement, the specimen’s dimensions were measured using a high-precision digital caliper with an accuracy of 0.001 mm. In the fracture test, two rounded supports were used, and the span was 20 mm. The fracture load was recorded in newtons (Figure S1). The flexural strength (σ) was calculated in megapascals and the flexural modulus was calculated in gigapascals using the following Equations (1) and (2):

(1) $$\sigma =3Fl/2bh^{2}$$

(2) $$E=Fl^{3}/4bh^{3}d$$

where *F* is the fracture load in newtons, *l* is the span between the supports in millimeters, *b* is the width of the specimen in millimeters, *h* is the height of the specimen in millimeters, and *d* is the deflection (in millimeters) corresponding to the *F*.

The Weibull characteristic strength (*σ*_0_) and the Weibull modulus (*m*) were calculated using the following Equation (3):

(3) $$P_{f}=1-exp\left[-{\left(\frac{\sigma }{\sigma_{0}}\right)}^{m}\right]$$

where *P_f_* is the probability of failure (between 0 and 1), σ is the flexural strength in megapascals, $\sigma_{0}$ is the Weibull characteristic strength in megapascals (the value at each 63.2% of the specimens fail), and *m* is the Weibull modulus.

### 2.3. Vickers Hardness Test

The Vickers hardness was measured using disc-shaped specimens with a diameter of 5 mm and a thickness of 2 mm. The size of the specimen was measured after post-curing process using a digital caliper with an accuracy of 0.01 mm. After storing the 3D printed specimen in a 37 °C incubator for 24 h, the hardness was measured using a micro Vickers hardness tester at 24.5°–25.5° (*n* = 5, indentation applied with a load of 300 g for 15 s; HMV-2, Shimadzu, Tokyo, Japan). Each specimen was measured three times on each of the upper and lower surfaces, with the mean value calculated as the result.

### 2.4. Evaluation of Color Change

The color change was evaluated according to the post-curing time after printing of the 3D printed resin by preparing five 10-mm-diameter and 2-mm-thick specimens in each group. The fabricated specimens were polished on both sides using carbon paper of up to 1200 grit under water cooling. The polished specimens were cleaned for 30 s in an ultrasonic cleaner, and the cleaned specimens were stored in distilled water at 37 °C for 24 h. A colorimeter (Cr321 Chromameter, Minolta, Osaka, Japan) was used to measure the color quantitatively, and three color measurements were performed for each specimen, with the mean value being recorded. L*, a*, and b* values measured at each time point were applied to the CIEDE2000 formula (ΔE_00_) to evaluate the change in color. The ΔE_00_ value was calculated using the following Formula (4):

(4) $$\Delta E_{00}=\;\sqrt{{\left(\frac{\Delta L}{K_{L}S_{L}}\right)}^{2}+{\left(\frac{\Delta C}{K_{C}S_{C}}\right)}^{2}+{\left(\frac{\Delta H}{K_{H}S_{H}}\right)}^{2}+R_{T}\left(\frac{\Delta C}{K_{C}S_{C}}\right)\left(\frac{\Delta H}{K_{H}S_{H}}\right)}$$

L*, a*, and b* values were taken on a white background, and the parametric values of *K_L_*, *K_C_*, and *K_H_* were set to 1. If the ΔE_00_ value did not exceed 2.25, the color change was assumed to be clinically acceptable.

### 2.5. Degree of Conversion

Fourier-transform infrared spectroscopy (FTIR) analysis was performed to identify the functional groups present in the 3D printed resin. Specimens with a diameter of 10 mm and a thickness of 2 mm were prepared, and the surface was polished with carbon paper of up to 1200 grit. The infrared spectrum for various materials and post-curing times were obtained using a Vertex 70 FTIR spectrometer (Bruker, Karlsruhe, Germany). The spectrum was recorded in absorbance mode using a diamond crystal plate, and obtained with a resolution of 4 cm^−1^ in the spectral region of 500–4000 cm^−1^. The experiment was performed three times for each of the evaluated groups. In each of the spectra, the heights of the absorption bands of the aliphatic and aromatic C=C bonds were measured at 1637 and 1608 cm^−1^, respectively. The degree of conversion (DC) was determined according to the following Equation (5):

(5) $$DC\;\left(\%\right)=\;\left\{1-\frac{1637\;{\mathrm{cm}}^{-1}/1608\;{\mathrm{cm}}^{-1}{}_{Peak\;height\;\left(cure\right)}}{1637\;{\mathrm{cm}}^{-1}/1608\;{\mathrm{cm}}^{-1}{}_{Peak\;height\;\left(uncure\right)}}\right\}\;\times 100$$

### 2.6. Biocompatibility Test

Cell viability and cytotoxicity analyses were performed to evaluate the biocompatibility of the 3D-printed resin according to their post-curing times. Specimens with a diameter of 10 mm and a thickness of 2 mm were prepared for the biocompatibility test. Specimens were 3D printed similar to that of the previous specimen production method, and post-curing was also performed similarly. The surface of the finished specimen was polished with 1200-grit carbon paper. Specimens were sterilized using ethylene oxide gas to prevent surface contamination.

Primary gingival fibroblasts (PCS-201-018) were purchased from the American Type Culture Collection (ATCC, Manassas, VA, USA). The cells were cultivated in Dulbecco’s modified Eagle’s medium (DMEM; WelGene, Daegu, Korea) supplemented with 10% fetal bovine serum (FBS; Thermo, Waltham, MA, USA) and penicillin/streptomycin (WelGene). The cells were incubated at 37 °C in 5% CO_2_, 95% air atmosphere, and 100% relative humidity. The cell culture medium was changed every 2–3 days. When the cells reached 85–90% confluency, they were treated with a trypsin-ethylenediaminetetraacetic acid (EDTA) solution (WelGene); the separated cells were resuspended in culture medium and seeded into new culture dishes at 1:5 dilution.

Cell viability was evaluated using a the CELLOMAX™ viability kit based on the tetrazolium salt (2-(2-methoxy-4-nitrophenyl)-3-(4-nitrophenyl)-5-(2,4-disulfophenyl)-2H-tetrazolium and monosodium salt [WST-8]; Precaregene, Hanam, Kyungido, Korea). First, circular 3D printing resin from each group was distributed in 48-well plates. Gingival fibroblasts were trypsinized and seeded into 48-well plates at a density of 5 × 10^4^ cells/well on each 3D printed resin sample. After culturing for 24 and 48 h, 50 μL of CELLOMAX™ solution was added to each well. After incubation at 37 °C for 2 h, the optical density (OD) at 450 nm was measured using a microplate reader (VersaMax Tunable microplate reader; Molecular Devices Co., Sunnyvale, CA, USA). Cell viability Equation (6):

(6) $$Cell\;viability\;\left(\%\right)=\;\left(\frac{O.D_{test\;sample}-O.D_{blank}}{O.D_{control}-O.D_{blank}}\right)\;\times 100$$

Cytotoxicity of the 3D printing resin on gingival fibroblasts was measured using lactate dehydrogenase (LDH) release assay (Quanti-LDH™ Cytotoxicity Assay Kit; BIOMAX, Seoul, Korea) according to the manufacturer’s protocol. Gingival fibroblast cells were seeded in a 48-well plate at a density of 5 × 10^4^ cells/well on each 3D printing resin. After 24 and 48 h, 10 μL of the culture medium were collected and centrifugated at 7000 rpm for 3 min. Thereafter, 10 μL of the supernatant was transferred to a new 96-well plate and 100 μL of LDH substrate mix was added. After 30 min of incubation at room temperature, the absorbance of the resultant solution was measured at 450 nm. Percentage cytotoxicity was calculated as follows Equation (7):

(7) $$Cytotoxicity\;\left(\%\right)=\;\left[\frac{\left(Cells\;with\;3D\;printed\;resin-Background\;control\right)-Low\;control}{High\;control-Low\;control}\right]\;\times 100$$

Background control: medium only; Low control: culture supernatants of fibroblast cells only; High control: supernatants of fibroblast cells after homogenization in cell lysis buffer.

All group data were verified as conforming to a normal data distribution (Kolmogorov–Smirnov test) and having a homogeneous variance (Levene test). One-way ANOVA was performed to test the significance of each material group according to post-curing time, followed by a post-hoc Bonferroni test. SPSS Statistics software (version 23.0, SPSS, Chicago, IL, USA) was used for all of the analyses, with the cutoff for significance set at α = 0.05.

## 3. Results

### 3.1. Flexural Strength Test and Weibull Analysis

**Figure 2:**
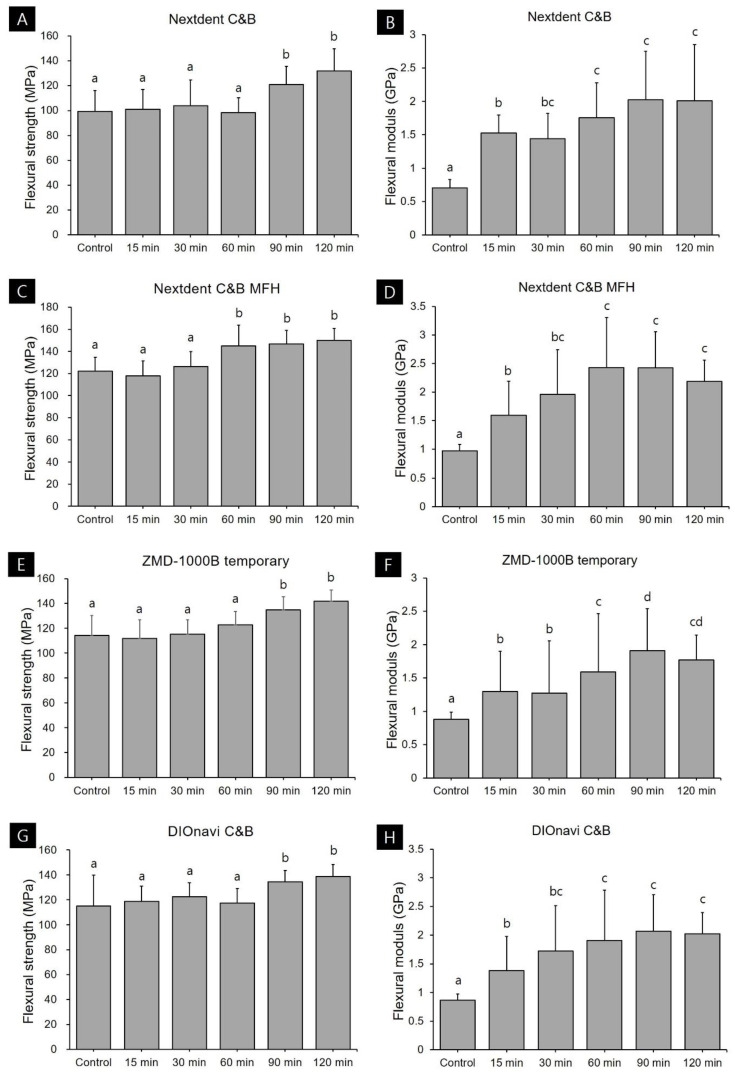
Flexural strength and modulus data according to post-curing time for each 3D printed resin: (**A**,**B**) Nextdent C&B, (**C**,**D**) Nextdent C&B MFH, (**E**,**F**) ZMD-1000B temporary, and (**G**,**H**) DIOnavi C&B. Data are mean and one-positive-standard-deviation values. Different lowercase superscript letters indicate significant differences in flexural strength and modulus for the same 3D printed materials.

Flexural strength data are presented in Table 2 and Figure 2. The flexural strengths of the four photopolymerized 3D printed resins used in this study increased with the post-curing time. In the case of Nextdent C&B resin, the control group in which post-curing was not performed after 3D printing exhibited a flexural strength of 99.29 MPa, while the values were 120.93 and 131.94 MPa in the groups that received 90 and 120 min of post-curing, respectively. This confirmed that the flexural strength increased significantly in the group that had undergone post-curing for more than 90 min. In the case of Nextdent C&B MFH resin, the flexural strength was 121.91 MPa in the group without post-curing, and there was no significant difference in strength between the 15- and 30-min groups. However, the flexural strength improved significantly in the group that had undergone post-curing for more than 60 min, to 144.99–150.04 MPa. For ZMD-1000B temporary resin, the flexural strength did not differ significantly between the group that did not undergo post-curing or had received less than 60 min of post-curing (111.80–122.69 MPa). However, the flexural strength improved significantly in the groups subjected to 90 and 120 min of post-curing, to 134.78 and 141.81 MPa, respectively. DIOnavi C&B resin also showed a significant increase in flexural strength (to 134.35–138.65 MPa) in the group that had undergone post-curing for more than 90 min.

Flexural modulus was significantly lower in the group without post-curing. Nextdent C&B, Nextdent C&B MFH, ZMD-1000B temporary, and DIOnavi C&B resins showed a flexural modulus of 0.71, 0.97, 0.88, and 0.86 GPa, respectively, and all of them were less than 1. In all the materials, the flexural modulus values gradually increased as the post-curing time increased. When the curing was performed for 120 min, the value increased to 1.77–2.08 GPa.

**Figure 3:**
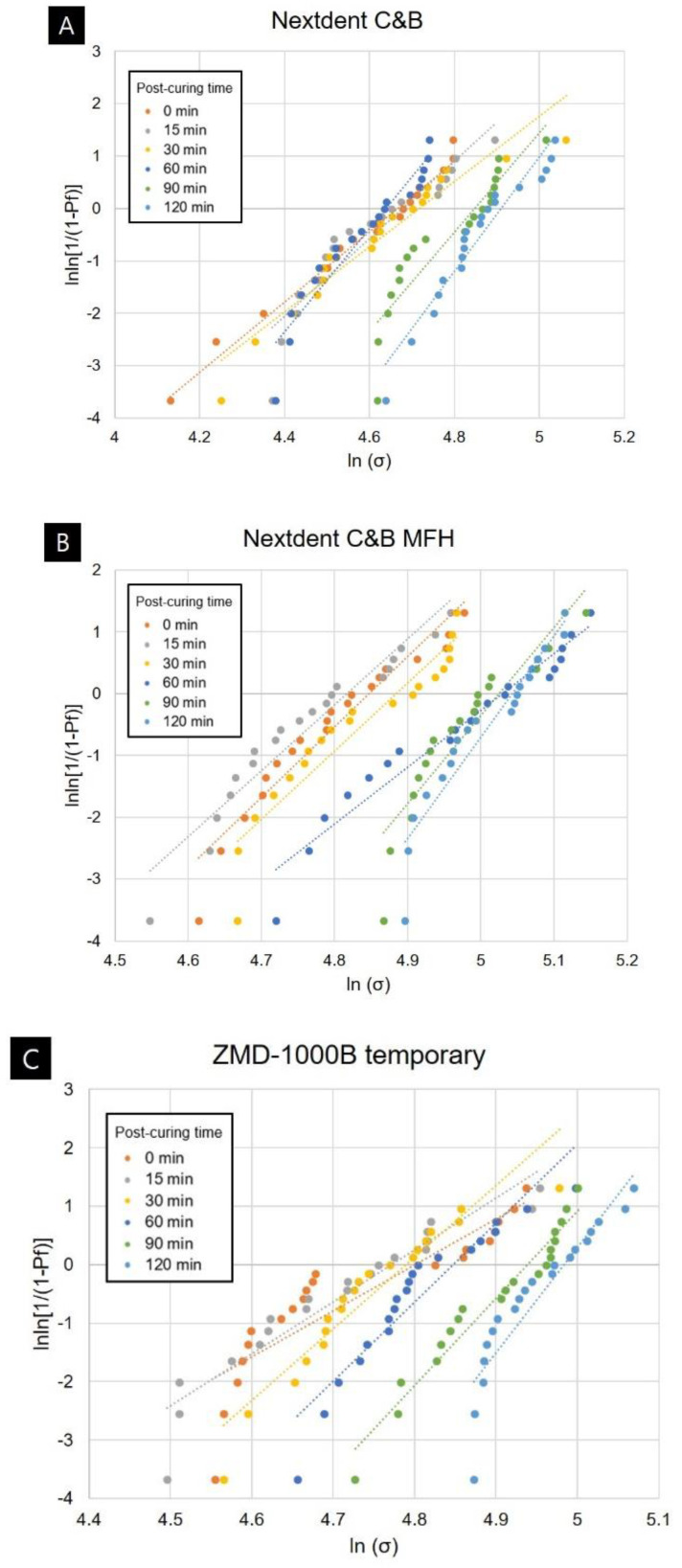

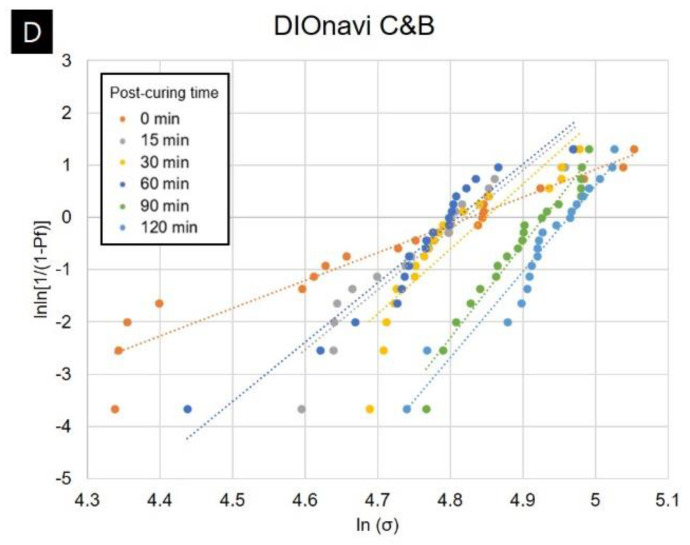
Weibull plots of 3D printed resin specimens for different post-curing times: (**A**) Nextdent C&B, (**B**) Nextdent C&B MFH, (**C**) ZMD-1000B temporary, and (**D**) DIOnavi C&B.

Weibull analysis was performed to obtain Weibull strength ($\sigma_{0}$) and Weibull modulus (*m*) data for each group. The results of the Weibull analysis are presented in Table 2 and Figure 3. The Weibull modulus showed a tendency to increase with the post-curing time for all four types of 3D printed resins used in this study. The values were 5.30–11.46 in the green-state group without post-cure, and 10.95–18.09 in the 120-min post-cure group.

### 3.2. Vickers Hardness Test

**Figure 4:**
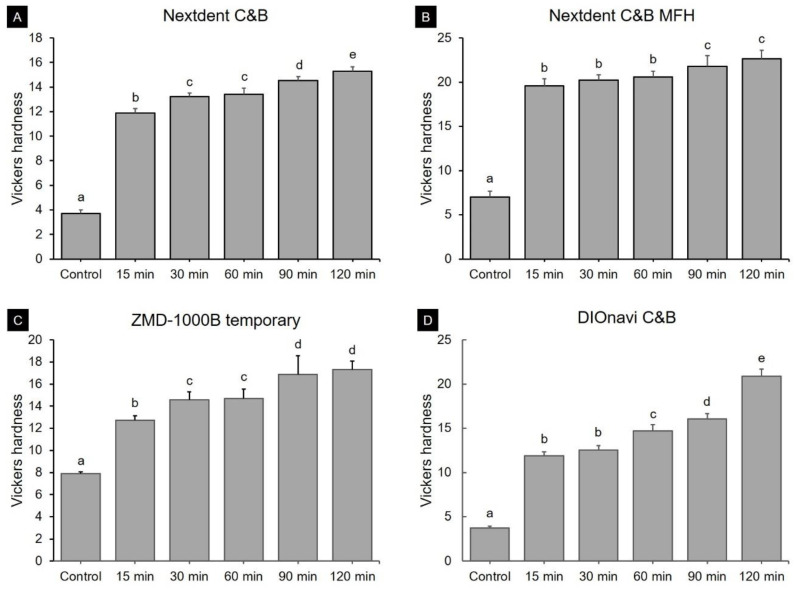
Vickers hardness according to post-curing time for (**A**) Nextdent C&B, (**B**) Nextdent C&B MFH, (**C**) ZMD-1000B temporary, and (**D**) DIOnavi C&B resins. Data are mean and one-positive-standard-deviation values. Different lowercase superscript letters indicate significant differences in Vickers hardness for the same 3D printed materials.

The results of the Vickers hardness test, which was conducted for all groups, are shown in Figure 4. The pattern of change was the same for all four materials. The Vickers hardness was as low as 3.70–7.90 in the green state, while it increased to 11.86–19.60 in the 15-min post-curing group, and to 15.29–22.66 in the 120-min post-curing group.

### 3.3. Evaluation of Color Change

**Figure 5:**
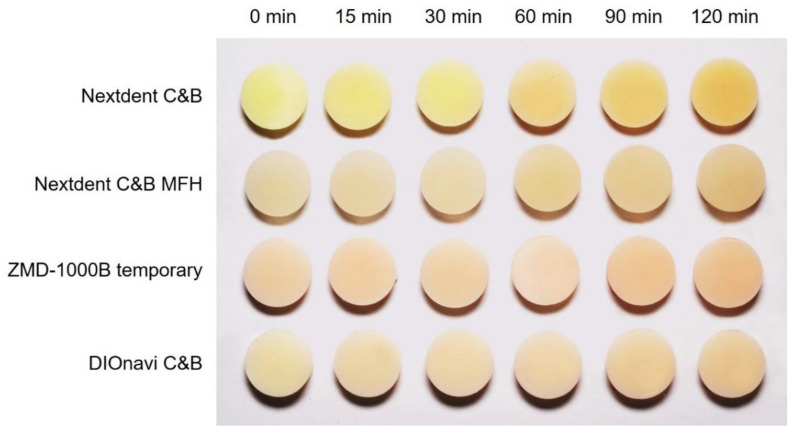
Photographs of the qualitative color changes of 3D printed resins, confirming that the light-yellow colors became somewhat darker and the reddish colors intensified with the post-curing time.

Figure 5 shows the overall pattern of qualitative color changes for the four resins used in this study. As the post-curing time increased, the light-yellow colors became a little darker and the reddish colors intensified.

**Figure 6:**
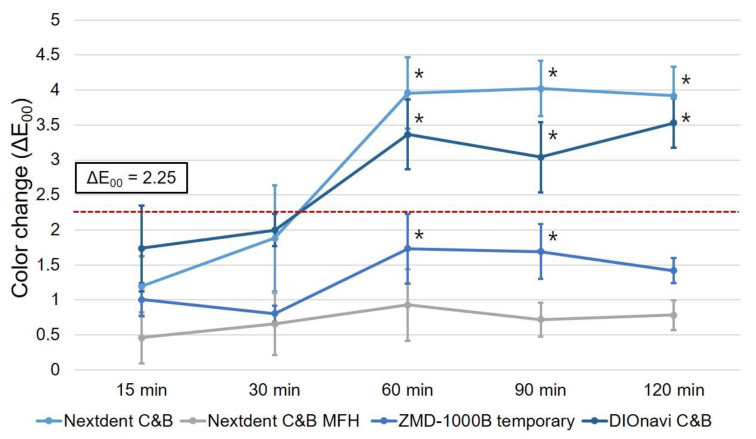
Color changes according to post-curing time of 3D printed resins relative to the control group without post-curing. No noticeable color change was observed until 15 and 30 min, while ΔE_00_ values of ≥2.25 were observed for Nextdent C&B and DIOnavi C&B resins after 60 min. Data are mean and standard-deviation values. Asterisk shows a statistically significant difference compared with the color change of the 15 min of post-curing specimens.

Based on the green-state group, the ΔE_00_ values calculated according to the post-curing time are shown in Figure 6. For Nextdent C&B MFH resin, no significant change in the ΔE_00_ value was observed in all groups after 15 to 120 min of post-curing. For the remaining three types of resins, the ΔE_00_ value changed significantly in the group subjected to post-curing for more than 60 min. In particular, in the case of Nextdent C&B and DIOnavi C&B resins, the ΔE_00_ value exceeded the clinically acceptable limit of 2.25 in most groups after at least 60 min of post-curing.

### 3.4. Degree of Conversion

**Figure 7:**
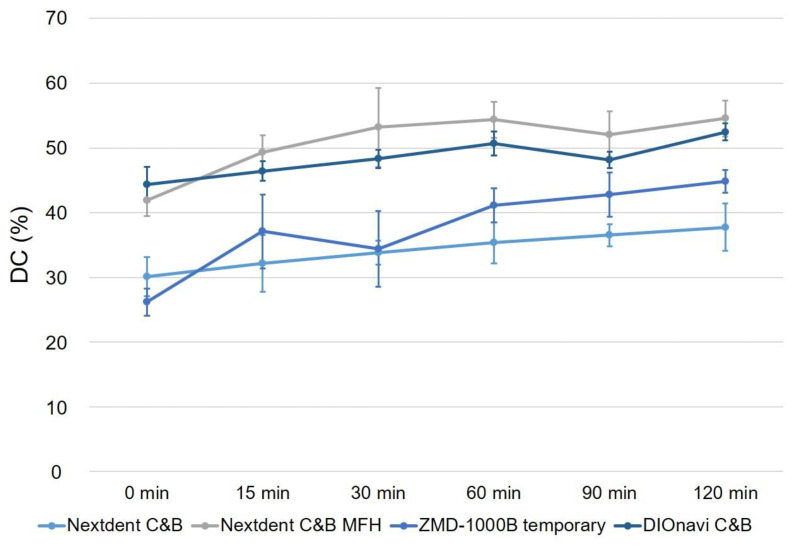
The DC value gradually increased with the post-curing time of the 3D printed resins. Data are mean and standard-deviation values.

The results for the DC are depicted in Figure 7. In all of the photopolymerized 3D printed materials used in this study, the polymerization rate increased with the post-curing time compared with that in the green-state group. The DC was 30.15–37.75% for Nextdent C&B resin. The DC was 41.95% in the green state and 54.55% after 120 min of post-curing for Nextdent C&B MFH resin, which represented the highest polymerization rate. The polymerization rate increased significantly with the post-curing time (*p* < 0.001). ZMD-1000B temporary resin showed a polymerization rate of 26.17% in the green state, which increased significantly to 37.09% after 15 min of post-curing. The polymerization rate tended to increase significantly with the post-curing time (*p* < 0.001). After curing for 120 min, the polymerization rate was 44.80%. The polymerization rate of DIOnavi C&B resin also increased significantly with the post-curing time (*p* < 0.001), and was 44.36–52.47%.

### 3.5. Biocompatibility Test

**Figure 8:**
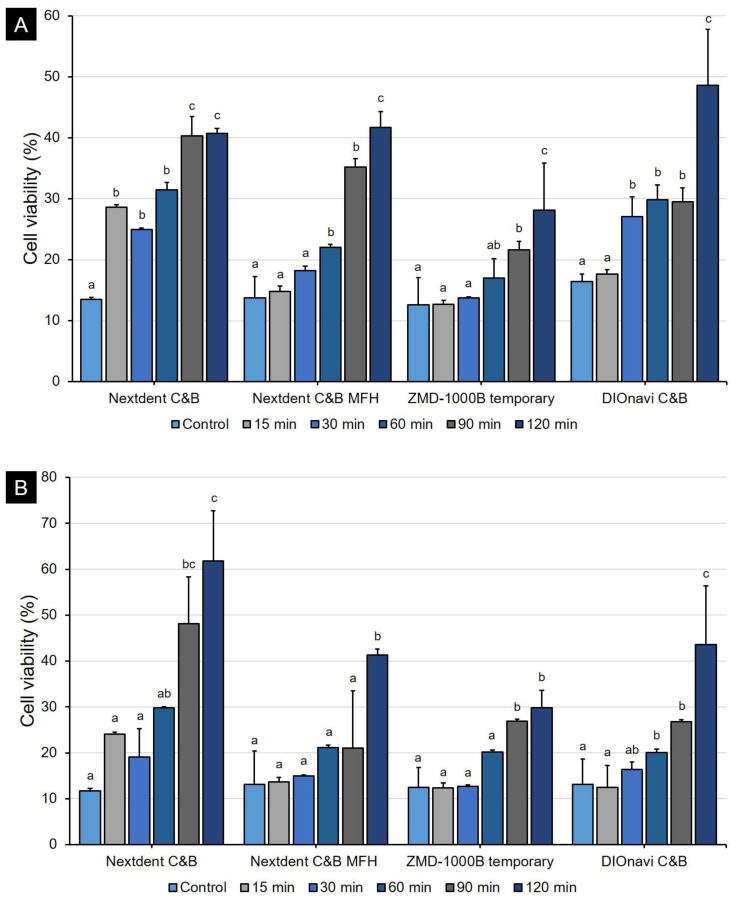
Cell viability evaluation graph by 3D printing resin. The cell viability gradually increased with the post-curing time. Data are shown as mean and standard deviation values. (**A**) 24 h and (**B**) 48 h. Different lowercase superscript letters indicate significant differences in cell viability for the same 3D printed materials.

**Figure 9:**
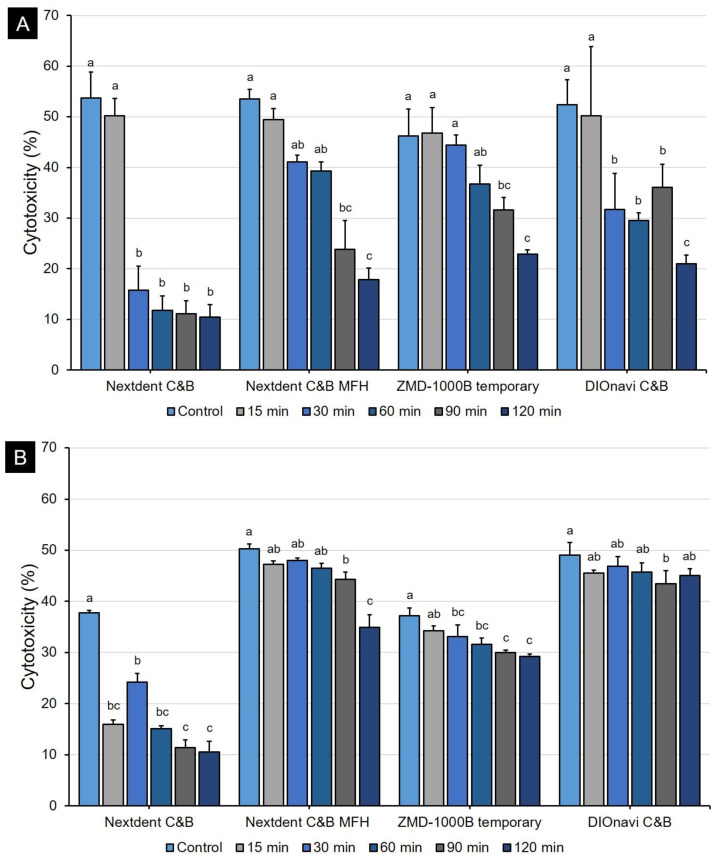
Evaluation of the cytotoxicity of 3D printing resins according to post-curing time. The cytotoxicity value gradually decreased with the post-curing time of the 3D printed resins. Data are mean and standard deviation values. (**A**) 24 h and (**B**) 48 h. Different lowercase superscript letters indicate significant differences in cytotoxicity for the same 3D printed materials.

The human gingival fibroblasts were cultured on 3D-printed samples. After 24 h, cell proliferation assay showed different results depending on the post-curing time for all kinds of 3D printing resins (Figure 8A). When post-curing was not performed, the cell viability was very low (about 12.6–16.4%). However, as the post-curing time increased, the cell viability also continuously increased. In the 90- and 120-min post-curing groups, the growth of cells was generally good, with cell viability of up to 48.6%. The 48-h result showed a similar pattern when compared with the 24-h result (Figure 8B). In the 24-h LDH cytotoxicity assessment, the cytotoxicity was very high (about 46.2–53.7%), but we found that the cytotoxicity continued to decrease as the post-curing time increased (Figure 9A). When curing was conducted for 120 min, we confirmed that the cytotoxicity was very low (about 10.4–22.9%). In conclusion, we observed that cytotoxicity tended to be inversely proportional to the results of WST-8 assay. The trend was the same even at 48 h, but the cytotoxicity was relatively high as compared to that at 24 h (Figure 9B).

## 4. Discussion

This study evaluated the properties of crown and bridge resin materials manufactured by 3D printing—a type of additive manufacturing—according to the post-curing time. Various physical properties of the produced samples differ between additive manufacturing and subtractive manufacturing in which premade CAD/CAM blocks are milled. Subtractive manufacturing involves making a prosthesis by milling a blank disk whose strength and color have already been determined, because the disk is manufactured in an environment of high temperature and high pressure in advance [29]. In contrast, additive manufacturing uses a photopolymerized resin to manufacture a prosthesis with a 3D printer, so that the results may vary if the parameters change during 3D printing and post-curing [14,16,17]. Optimal parameter settings are therefore required when using 3D printers and photopolymerized resins, and clinicians and dental technicians should be provided with appropriate scientific guidelines. The present study found that the flexural strength, hardness, and degree of conversion increased with the post-curing time of the 3D printed crown and bridge resin materials produced using 3D printing technology, and significant changes in color were observed. Therefore, the null hypothesis of this study was rejected.

This study confirmed that the flexural strength of the 3D printed objects improved significantly when post-curing was performed for more than 60 or 90 min depending on the type of 3D printed photopolymerized resin. No improvement in flexural strength was apparent when the post-curing time was shorter. This is thought to be due to increased brittleness resulting from the elasticity of the material decreasing, rather than the flexural strength improving with the degree of conversion of the most-superficial layer [30,31,32]. This can also be confirmed by the fact that the results of flexural modulus analysis showed that the group that did not undergo post-curing had a very low value, and that the value increased as post-curing progressed. It seems that anisotropy can be resolved and mechanical properties can be improved when a sufficient post-curing time is applied to achieve homogeneous polymerization in both deep and superficial layers of the 3D printed object [33,34]. The Weibull modulus is an indicator of the structural homogeneity and strength of a material [35]. The Weibull analysis in this study also confirmed that the mechanical strength and reliability improved with the post-curing time for all 3D printed resins. The Weibull modulus of ZMD-1000B temporary resin increased for post-curing times of more than 30 min, that of Nextdent C&B resin increased for post-curing times of 60 min or more, and those of Nextdent C&B MFH and DIOnavi C&B resins increased when post-curing was performed for more than 90 min. It is considered that a sufficient post-curing time is essential to improve the reliability of a 3D printed prosthesis [36]. Other studies evaluating the flexural strength of 3D printed resins according to the post-curing time only applied post-curing times of up to 40 min [37]. Bonada et al. [37] evaluated the mechanical strength of photopolymer resin according to the post-curing time. A flexural strength of 28.87 MPa was reported for their group without post-curing, while it was 41.72 MPa for their group after 20 min of post-curing. The group that underwent 40 min of post-curing showed 40.36 MPa, which did not differ from the flexural strength in the group with 20 min of post-curing. That study found a significant difference in flexural strength at 20 min, which is the initial post-curing period, with no change in flexural strength occurring thereafter. Those results contrast with the present findings of improvements in mechanical properties for a post-curing time of 60 or 90 min. These discrepancies between the previous and the present study might have been due to differences in various processes and Bonada et al.’s study used clear resins that facilitate light transmission during the 3D printing process and the post-curing process. However, the range of flexural strengths found in that study was 28.87–41.72 MPa, which differs markedly from the flexural strengths of 80–130 MPa that are generally reported for 3D printed crown and bridge resins [38]. Unkovskiy et al. [11] evaluated changes in the flexural strength of 3D printed crown and bridge resins when post-curing was performed for 15 min using three types of post-curing equipment. That study found flexural strengths of 128.36–137.43 MPa for the resins post-cured for 15 min, which represent significant improvements over the flexural strength of 47.31 MPa for the green-state resin without post-curing. In our study the flexural strength did not differ significantly between the green-state group that did not undergo post-curing and the group that underwent 15 min of post-curing. Therefore, the results of these two studies also differed, which would have been due to differences in the composition of the material used in the 3D printing process, different parameters such as the exposure time or the thickness of each layer in the 3D printing process, and differences in the intensity of the 3D printer light source resulting in differences in the mechanical properties of the green-state group that has just been printed. Reymus et al. [23] analyzed the differences in fracture strength according to post-curing equipment and resin types. They found that the outcome of the post-curing process was significantly affected by the type of post-curing equipment and resin, with there also being an interaction between these two factors. Together these observations confirm that various factors besides the post-curing time affect the mechanical properties of a 3D printed resin. However, a sufficient post-curing time of 60 min or more resolves the anisotropy of the 3D printed object and improves its physical properties.

It is known that the color tone of 3D printed resin varies greatly between the available commercial products, and that the color dimension differs markedly from that of conventional PMMA resin [13]. The present study found many differences in color dimensions were identified qualitatively. In particular, as the post-curing time increased, the overall red color gradually intensified (Figure 6). It is known that changes in the color tone with the curing time are mainly due to the photoinitiator [39,40]. An appropriate combination of photoinitiator and coinitiator, as well as the correct exposure time to the light source can cause desirable changes in color tone and improve both the biocompatibility and mechanical strength [26]. Various types of photoinitiator are used, includin diphenyl (2,4,6-trimethylbenzoyl) phosphine oxide (TPO), bis phenyl (2,4,6-trimethylbenzoyl) phosphine oxide (BAPO), camphorquinone, and 2-(dimethylamino) ethyl methacrylate. It is known that the amount of yellowing is greater when TPO and BAPO are used as photoinitiators in the process of photopolymerization [40,41]. It is also known that these two photoinitiators polymerize rapidly, and colored peroxide is formed due to an increase in temperature during polymerization, with significant yellow discoloration occurring [41]. The color tone can also change with the photoinitiator concentration, and a high photoinitiator concentration will increase the absorption of light energy, which will in turn greatly influence the expression of color [41]. It therefore seems that the color change with the post-curing time was also affected by the type and concentration of the photoinitiator of the four 3D printed resins used in this study. It is also possible that the color tone of 3D printed resin can change with the characteristics of light source of the post-curing equipment. Reymus et al. [24] found that post-curing Nextdent C&B resin with Otoflash or Labolight DUO equipment did not result in the resin having the desired tooth color, whereas the tooth color was appropriately reproduced when the Printbox post-curing device was used. This was attributed to the pigment chemistry of Nextdent C&B resin and the light source of the Printbox device being well matched. For the 3D printed resins used in our study, there is a possibility that the pattern of color tone will differ with their combination with 3D printers or post-curing equipment.

The Vickers hardness tests revealed marked improvements compared with the green-state group after the initial post-curing time (15 min), which was not the case for the flexural strength in all of the 3D printed resin groups. After 15 min, the Vickers hardness gradually increased with the post-curing time, but not by much. The improvement in the hardness after a short post-curing time is probably attributable to the physical properties of the most-superficial layer first improving at the start of the post-curing treatment. This characteristic was similarly reflected in the results obtained for the degree of conversion. Since the degree of conversion evaluates the polymerization of the most-superficial layer, there was a tendency for the degree of conversion to improve slowly during post-curing after the DC had significantly increased in the specimens in the green state. Aromaa et al. [42] analyzed the hardness and DC of resin according to the post-curing time from the green state to 6 h later. Those authors reported that the DC increased very slowly during the post-curing process for up to 6 h, after an initial rapid increase. This pattern seems very similar to the findings of the present study. Aromaa et al. [42] found that the hardness of the surface increased rapidly between 2 and 6 h, which is thought to be attributable to resolution of the anisotropy of resin materials, similar to what our measurements of the flexural strength revealed. Reymus et al. [24] compared the degree of conversion of 3D printed objects between using four different post-curing devices. They found that the type of equipment markedly influenced the degree of conversion. Steyrer et al. [43] studied the degree of conversion in photopolymerized resins in which various types of photoinitiators were added at different ratios. They found that the polymerization rate of the resin increased with the photoinitiator concentration when the latter remained low, but then decreased at higher concentrations due to light transmission being hindered. The biocompatibility test conducted in our study showed a similar trend overall. When post-curing was not performed or was only performed for a very short time, the cell viability was low and cytotoxicity was high. Thus, it was confirmed that sufficient post-curing time was required to manufacture a biologically safe prosthesis. Distinctively, the cytotoxicity at 48 h was higher than that at 24 h. The reason for the increase in toxicity at 48 h could be the following: first, the effect of dead cells at 24 h, and second, the effect of residual monomers of 3D printing resin on the cells.

Insufficient resin polymerization results in deterioration of properties over time and insufficient long-term stability [20,44]. Most photopolymerized resins are based on (meth) acrylates, and polymerization involves the free radicals produced by photoinitiators: when the photoinitiator is exposed to an appropriate light source, it forms radicals and photopolymerization begins [43]. The gel point of a photopolymerization polymer can be reached even with a very low rate of polymerization and does not cause problems in the 3D printing process [43,45]. However, after 3D printing is completed, physical properties such as the mechanical strength are often insufficient, and so it is very important to improve the mechanical properties by resolving anisotropy and increasing the polymerization rate through the post-curing process after 3D printing [33,36]. However, as confirmed in this study, since an increase in the degree of polymerization does not occur across the entire cross-section of a sample, it does not necessarily have a positive effect on the physical strength. Addressing the situation where the degree of polymerization is increased only in the superficial layers and the overall strength might not be improved requires a sufficient post-curing time [43,46]. In addition, many companies that produce 3D printed resins recommend the most-appropriate combination of 3D printer and post-curing machine for increasing the likelihood of obtaining optimal results. However, the wide variety of 3D printers and post-curing equipment available commercially makes it difficult for all clinicians to access the several types of equipment that are suitable for various resins. Therefore, it is believed that trial and error through repeated pilot tests is required to obtain the optimal properties when using the specific equipment available to clinicians or dental technicians, and also a sufficient post-curing time is required to further improve the properties of 3D printed objects.

This study had several limitations. First, the composition of 3D printed resin could not be analyzed in detail. Although 3D printed resins generally comprise photoinitiators, mono- or multifunctional monomers, and functionalized oligomers, the exact composition of each resin is not disclosed due to patent rights and priority rights [47]. More in-depth analyses will be possible if detailed information on the compositions of the resins can be obtained in the future. Second, only the DC for the superficial layers of 3D printed object was evaluated in the present study. The specimens were about 2 mm thick, the overall anisotropy was resolved, and the mechanical strength was improved when the post-curing time was long. Therefore, observing the DC changes in both the deep and superficial layers would improve the understanding of the overall post-curing process. Although 3D printing is now very common and its application has expanded in the dental field, there has been relatively little research into the optimization of 3D printing processes related materials. There are many types of 3D printers and associated differences in their underlying technologies, as well as many parameters that affect the quality of printed objects. In-depth research in these fields will increase the reliability and predictability of dental treatment processes that involve the use of 3D printed crown and bridge materials [15].

## 5. Conclusions

Within the limitations of this study, the following conclusions can be drawn. Physical properties such as the flexural strength, Weibull modulus, surface hardness, and degree of conversion have been improved in the 3D printing and post-curing processes using four types of 3D printed resins (Nextdent C&B, Nextdent C&B MFH, ZMD-1000B temporary, and DIOnavi C&B). In addition, it was confirmed that the color tone of the 3D printed crown and bridge resin differ significantly with the post-curing time. In particular, the large diversity of the characteristics of 3D printers and post-curing equipment utilized by clinicians and dental technicians means that a sufficient post-curing time should be applied in order to achieve reliable clinical results when using 3D printed resins. Further studies of the various parameters influencing the 3D printing process and post-curing process are needed to obtain more clinically meaningful results.

## Figures and Tables

**Table 1:** CAD/CAM milling blocks and 3D printed resins used in this study.

Product	Components	Manufacturer
Nextdent C&B	>90% Methacrylic oligomers, methacrylate monomer, <3% phosphine oxides, pigment	Nextdent, Soesterburg, The Netherlands
Nextdent C&B MFH	Methacrylic oligomers, methacrylate monomer, inorganic filler, phosphine oxides, pigment	Nextdent, Soesterburg, The Netherlands
ZMD-1000B temporary	Urethane methacrylate oligomer, acrylate monomer, methacrylate monomer, phosphine oxide, cerium oxide, titanium oxide	Dentis, Daegu, Korea
DIOnavi C&B	>90% Methacrylic oligomers, <10% phosphine oxides, pigment	DIO Incorporated, Busan, Korea

**Table 2:** Weibull strength ($\sigma_{0}$) and Weibull modulus (*m*) data for the four types of 3D printed resin specimens according to post-curing time.

Specimen	Post-Curing Time	m	σ_0_, MPa
Nextdent C&B	Control	6.71 (6.23–7.19)	106.37
	15 min	7.42 (6.01–8.82)	107.40
	30 min	6.21 (5.35–7.07)	111.79
	60 min	9.82 (8.35–11.29)	103.39
	90 min	9.49 (7.53–11.45)	127.52
	120 min	10.95 (9.43–12.47)	135.58
Nextdent C&B MFH	Control	11.46 (9.89–13.02)	127.17
	15 min	10.69 (9.25–12.12)	123.32
	30 min	11.05 (9.17–12.93)	132.18
	60 min	9.20 (8.17–10.23)	152.83
	90 min	14.24 (11.41–17.07)	152.11
	120 min	16.43 (13.80–19.06)	154.87
ZMD-1000B temporary	Control	7.87 (5.81–9.92)	121.26
	15 min	8.88 (7.51–10.25)	117.95
	30 min	12.25 (10.24–14.25)	120.03
	60 min	13.54 (11.18–15.90)	127.37
	90 min	14.91 (13.23–16.59)	139.44
	120 min	18.09 (14.17–22.00)	145.81
DIOnavi C&B	Control	5.30 (4.57–6.03)	125.00
	15 min	11.48 (9.67–13.30)	124.25
	30 min	12.42 (9.18–15.66)	127.67
	60 min	11.35 (9.61–13.09)	122.91
	90 min	17.84 (15.87–19.8)	138.14
	120 min	16.44 (14.43–18.46)	143.18

## Supplementary Materials

The following are available online at https://www.mdpi.com/2073-4360/12/11/2762/s1, Figure S1: Representative curves of fracture load of the 3D printing resin (DIOnavi C&B) used in this study. It can be observed that the fracture load continues to increase as the post-curing time increases, and the shorter the post-curing time, the greater the displacement.
