# Supplementary material for: Effects of Post-Curing Time on the Mechanical and Color Properties of Three-Dimensional Printed Crown and Bridge Materials
Source: Polymers (Basel). 2020 Nov 23;12(11):2762. doi: 10.3390/polym12112762 (PMC7700600; doi:10.3390/polym12112762)
Supplement: Supplementary file 1 [file polymers-12-02762-s001.pdf]

Supporting information

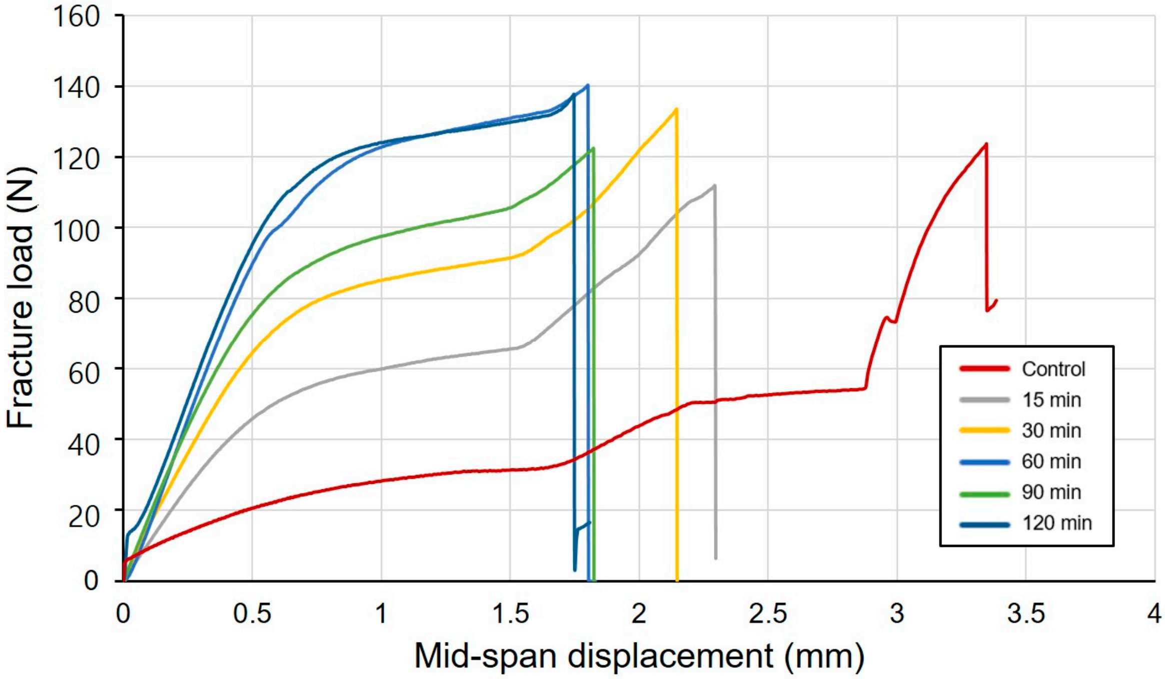

Figure S1. Representative curves of fracture load of the 3D printing resin (DIOnavi C&B) used in this study. It can be observed that the fracture load continues to increase as the post-curing time increases, and the shorter the post-curing time, the greater the displacement.
